# The Surgical Treatment of Irremovable Cancer of the Uterus

**Published:** 1902-08-16

**Authors:** 


### THE SURGICAL TREATMENT OF IRREMOVABLE CANCER OF THE UTERUS.

At the end of an interesting paper on Operations for Cancer of the Uterus, Dr. W. J. Sinclair[1] discusses the question whether we possess any surgical means of giving relief to the symptoms, or perchance checking the development of the disease in cases in which the malady has already gone so far as to make it impossible to hold out any hope of cure. He justly says that inasmuch as the great majority of cases when seen for the first time by the gynæcologist already belong to the category of the inoperable, palliative treatment becomes one of the most important chapters of the subject under discussion. In regard to this matter he says that the only surgical proceedings that are resorted to with confidence and with a certain amount of success are the use of the curette, or sharp spoon, followed by the actual cautery, or by the application of a very strong solution of chloride of zinc. All other escharotics appear to have been found ineffective or inconvenient. Dr. Sinclair prefers the zinc. The patient should be under an anæsthetic. It is assumed that the uterus is more or less fixed, and beyond the scope of any radical operation. With the parts exposed, preferably by means of the broad-bladed weighted speculum, the friable tissue produced by the ulcerating cancer is rapidly cleared out. Then the small pockets and cavities containing friable tissue are searched for, and all that is possible removed without the violent use of the curette. The finger may from time to time be passed into the cavity. In most cases considerable shreds and fragments of firmer tissue, which cannot be easily removed by the curette, remain projecting into the cavity. These should be quickly separated from their attachments by means of scissors. The cavity is now to be packed firmly with dry lint while the operator sees that his solutions are so arranged that they can be applied with precision. If a strong solution of chloride of zinc (1 in 2 or 3) is to be employed it should be applied to the cavity by soaking the end of a long shred of lint, the dry part of which can be pushed in to prevent the surplus fluid from flowing over into the vagina. Immediately after pledgets of cotton wool soaked in carbonate of soda solution or impregnated with the dry powder may be applied to the mouth of the cavity in the vagina. After the first effervescence is over these pledgets may be removed, and the whole vagina be packed with a long shred of lint slightly wrung out of a strong solution of carbonate of soda. The uterine and vaginal tampons may be removed after a few hours, and the flow of serum encouraged for a few days by hot boracic douches. The results are in some cases surprisingly satisfactory.

[1] Practitioner, July.

